# The bioelectric well: a novel approach for *in situ* treatment of hydrocarbon‐contaminated groundwater

**DOI:** 10.1111/1751-7915.12760

**Published:** 2017-07-11

**Authors:** Enza Palma, Matteo Daghio, Andrea Franzetti, Marco Petrangeli Papini, Federico Aulenta

**Affiliations:** ^1^ Department of Chemistry – Sapienza University of Rome P.le Aldo Moro 5 Rome 00185 Italy; ^2^ Water Research Institute (IRSA) – National Research Council (CNR) Via Salaria km 29 300 Monterotondo (RM) 00015 Italy; ^3^ Department of Earth and Environmental Sciences – University of Milano‐Bicocca Piazza della Scienza 1 Milan 20126 Italy

## Abstract

Groundwater contamination by petroleum hydrocarbons (PHs) is a widespread problem which poses serious environmental and health concerns. Recently, microbial electrochemical technologies (MET) have attracted considerable attention for remediation applications, having the potential to overcome some of the limiting factors of conventional *in situ* bioremediation systems. So far, field‐scale application of MET has been largely hindered by the limited availability of scalable system configurations. Here, we describe the ‘bioelectric well’ a bioelectrochemical reactor configuration, which can be installed directly within groundwater wells and can be applied for *in situ* treatment of organic contaminants, such as PHs. A laboratory‐scale prototype of the bioelectric well has been set up and operated in continuous‐flow regime with phenol as the model contaminant. The best performance was obtained when the system was inoculated with refinery sludge and the anode potentiostatically controlled at +0.2 V versus SHE. Under this condition, the influent phenol (25 mg l^−1^) was nearly completely (99.5 ± 0.4%) removed, with an average degradation rate of 59 ± 3 mg l^−1^ d and a coulombic efficiency of 104 ± 4%. Microbial community analysis revealed a remarkable enrichment of *Geobacter* species on the surface of the graphite anode, clearly pointing to a direct involvement of this electro‐active bacterium in the current‐generating and phenol‐oxidizing process.

## Introduction

Subsurface contamination by spilled PHs is a widespread problem, which poses serious environmental and health concerns (Poulsen *et al*., 1992; Durmusoglu *et al*., 2010). Bioremediation is typically regarded as a viable and sustainable technique to remediate PH‐contaminated sites, which exploits the vast metabolic diversity of microorganisms that use contaminants as carbon and energy sources in their metabolism (Atlas, 1995). The majority of PHs are biodegradable under aerobic conditions; therefore, typical bioremediation strategies involve oxygen delivery into the contaminated matrix to enhance the metabolism of naturally occurring aerobic hydrocarbon‐degrading microbial populations (Farhadian *et al*., 2008). Although widely applied, this strategy suffers of a number of limitations such as (i) the high energy consumption associated with aeration, (ii) the low efficiency of oxygen utilization due to the rapid consumption by reduced mineral substances such as Fe(II) and HS^−^, (iii) the undesired stripping of volatile contaminants and (iv) the high growth yield of aerobic microorganisms which may cause clogging problems near air/oxygen injection points (Boopathy, 2000; Höhener and Ponsin, 2014).

In recent years, microbial electrochemical technologies (MET) have attracted considerable attention for remediation applications. MET are anaerobic systems in which microorganisms catalyse oxidation or reduction reactions using solid‐state electrodes, suitably deployed in the contaminated matrix, as virtually inexhaustible electron acceptors or donors, respectively (Aulenta *et al*., 2009; Zhang *et al*., 2010, 2013; Lovley *et al*., 2011; Rodrigo Quejigo *et al*., 2016; Lai *et al*., 2017). Laboratory‐scale studies have shown that MET can be employed to stimulate the anaerobic oxidation of a variety of reduced contaminants in soil and groundwater, including lower chlorinated compounds and PHs (Daghio *et al*., 2017). In principle, MET have several potential advantages compared with conventional aerobic bioremediation strategies, such as (i) the possibility to promote the complete oxidation of contaminants with no need for adding oxygen or other electron acceptors [e.g. nitrate or soluble Fe(III) species]; (ii) the possibility to colocalize the microorganisms and the electron acceptor (i.e. the electrode) as well as (iii) the possibility to drive, control and monitor the biodegradation reaction (in the subsurface) with electrochemical means (Daghio *et al*., 2017). Collectively, all these aspects have the potential to dramatically increase the reliability, predictability and in turn applicability of *in situ* bioremediation systems. In spite of their promise, however, field‐scale applications of MET for subsurface remediation are still hindered by the poor availability of scalable bioelectrochemical reactor configurations that are amenable for *in situ* applications (Zhang and Angelidaki, 2013; Wang *et al*., 2015; Nguyen *et al*., 2016). Indeed, while few MET designs have been proposed to treat contaminated soils (Lu *et al*., 2014; Mao *et al*., 2016), very limited information is available on systems designed for the treatment of contaminated aquifers, possibly containing contaminants as a (lighter or denser) separate non‐aqueous phase. In this framework, the aim of this study was to describe and asses the bioremediation potential of a novel bioelectrochemical reactor configuration, hereafter named ‘the bioelectric well’, which can be installed directly within a groundwater well and that can be applied for *in situ* treatment of organic contaminants, such as PHs.

## Results and discussion

### Phenol degradation and electric current generation

**Figure 1:**
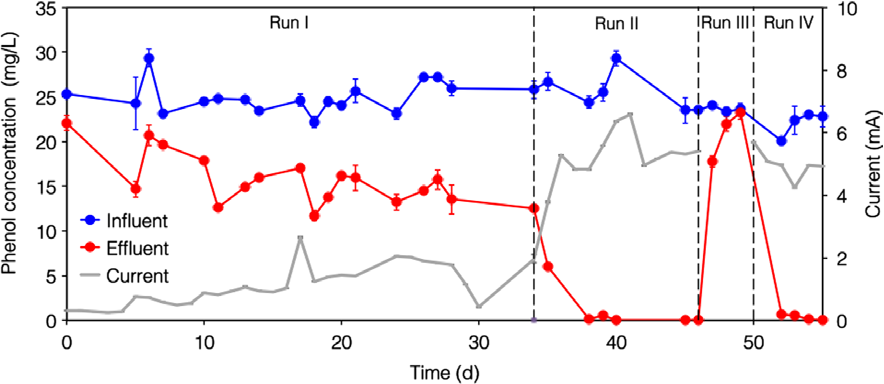
Performance of the bioelectric well throughout the experimental period.

The laboratory‐scale bioelectric well was operated continuously for a period of 56 days, corresponding to 128 hydraulic retention times (Fig. 1). During Run I (i.e. days 0−33), phenol removal gradually increased from 12% to approximately 50%. Accordingly, electric current generation slowly increased from 0.3 mA to approximately 1.9 mA. On average, phenol removal rate and coulombic efficiency were 23 ± 1 mg l^−1^ d and 72 ± 8% respectively (Table 1).

The performance of the system remarkably improved following bioaugmentation with a small volume (25 ml) of refinery wastewater. Indeed, effluent phenol concentration dropped from 9 mg l^−1^ (on day 33) to below instrumental detection limits, by day 40. Correspondingly, the average current output (5.3 ± 0.2 mA) and coulombic efficiency (104 ± 4%) were also markedly higher compared with Run I. Coulombic efficiency values exceeding 100% are most likely due to electron recycling effects, involving the anodic (bio)electrochemical oxidation of the hydrogen produced at the cathode, as also previously reported in the literature (Villano *et al*., 2011).

These findings suggest that the refinery wastewater harboured microorganisms capable of anaerobically oxidizing phenol using the graphite granules as terminal electron acceptors. Notably, the high value of coulombic efficiency observed during this run is consistent with phenol being completely oxidized to carbon dioxide and water. This hypothesis was also supported by measured values of the total organic carbon in the reactor effluent, which remained stably <5 mg l^−1^ during Run II. Notably, the herein obtained coulombic efficiencies are among the highest reported in the literature with phenol as the sole carbon and energy source, both in pure and mixed culture studies (Luo *et al*., 2009; Friman *et al*., 2013).

From day 46 to 49 (Run III), the bioelectric well was maintained at open circuit. The effluent phenol concentration rapidly increased and equalled the influent value on day 49. On average during Run III, the phenol removal rate was 2 ± 1 mg l^−1^ d, hence providing a further confirmation that phenol removal during Run I and Run II was directly linked to electric current generation. On day 50 (Run IV), the electrical continuity was re‐established, with the anode potentiostatically set at +0.2 V versus SHE. Consequently, phenol degradation rate, electric current and coulombic efficiency rapidly resumed to values that were similar to those observed during Run II, thereby indicating that the system was resilient to short periods of starvation (Table 1).

**Table 1:** Performance of the bioelectric well during continuous‐flow operation under different conditions. For each run, average values (and associated standard error) of relevant parameters were calculated from data collected after the system had been operated for at least three hydraulic retention times

Run	Day	Inoculum	Anode potential (V versus SHE)	Average phenol removal rate (mg l^−1^ d)	Average current (mA)	Average coulombic efficiency (%)
I	0–33	Municipal Activated sludge	+0.2	23 ± 1	1.2 ± 0.1	72 ± 8
II	34–45	Refinery wastewater	+0.2	59 ± 3	5.3 ± 0.2	104 ± 4
III	46–49	Refinery wastewater	OCP	2 ± 1	N.A.	N.A.
IV	50–56	Refinery wastewater	+0.2	53 ± 1	4.8 ± 0.2	108 ± 6

During experimental runs with the anode potentiostatically set at +0.2 V versus SHE, methane was stably detected in the headspace of the sampling cell in the outlet of the bioelectric well, consistently with hydrogen evolution likely being the predominant reaction occurring at the stainless steel cathode. Notably, the rapid conversion of abiotically produced hydrogen (which never accumulated within the system) into methane gas was essential to prevent pressure build‐up within the system.

Finally, throughout the study, oxygen was never detected either in the headspace of the bioelectric well or in the headspace of the influent and effluent sampling cells.

### Characterization of the bioelectric well microbial communities

**Figure 2:**
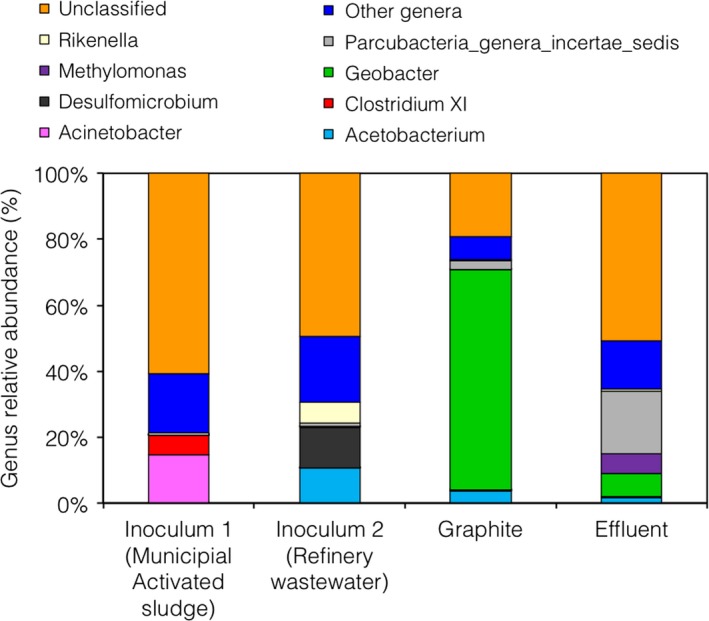
Taxonomic composition of the bacterial communities at the genus level. Only the genera with abundance of 5%, or higher, in at least one sample have been reported.

At the end of Run IV, DNA was extracted from the graphite granules and from the effluent of the reactor. The bacterial communities were characterized by sequencing of the 16S rRNA gene and compared with those of the municipal activated sludge (Inoculum 1) and of the refinery wastewater (Inoculum 2) used as inoculum at the start of Run I and Run II, respectively. The bacterial diversity was lower on the graphite (*H*′ = 0.9) compared with the microbial inocula (Inoculum 1, *H*′ = 2.8; Inoculum 2, *H*′ = 2.6) and to the effluent (*H*′ = 2.3). In particular, members of the genus *Geobacter* were highly enriched on the graphite (67% of the bacterial sequences) and were detected also in the effluent of the reactor (Fig. 2). This result is consistent with previous findings reporting the enrichment of *Geobacter* species at the anode of microbial fuel cells treating phenolic wastewater (Zhang *et al*., 2017) and with phenol being individuated as a key metabolic intermediate during benzene degradation by *Geobacter metallireducens* (Zhang *et al*., 2013).

The most abundant OTUs enriched on the graphite were OTU_1 and OTU_3 (36% and 29% respectively). Both OTU_1 and OTU_3 were classified within the genus *Geobacter* (80% confidence) and were observed with a lower relative abundance (~1%) in the effluent of the reactor. Sequences of same OTUs (~0.2%) were detected in the refinery wastewater (Inoculum 2) while only a low amount (OTU_1 < 0.001%), or no sequences (OTU_3), were detected in the municipal activated sludge (Inoculum 1). The enrichment of *Geobacter* on the graphite can thus be likely attributed to the addition of the refinery wastewater at the start of Run II, although it remains unclear which microorganisms were responsible for the initial slow bioelectrochemical oxidation of phenol during Run I.

### Implications for *in situ* treatment of contaminated aquifers

The herein described bioelectric well has specific design and functional characteristics which hold promise for a potential field implementation of this bioremediation technology. To start with, the concentric geometry of the system, with the cathode positioned in very close proximity of the granular anode, allows minimizing voltage and in turn energy losses. As an example, during sustained operation under optimal conditions (i.e. Run II), cell voltage remained stably below 0.90 V, which would in turn correspond to an electric energy consumption associated with contaminant degradation of 0.007 kWh per g of phenol removed (not accounting for the energy associated with groundwater recirculation). This value is extremely low, especially if compared with other (bio)treatment technologies, such as biosparging (Suthersan, 1997). Importantly, with the proposed system architecture, the distance between anode and cathode is not expected to increase substantially upon scaling up. Furthermore, it should also be noted that this energy consumption was associated with values of phenol degradation rates and electric current as high as 59 mg l^−1^ d and 5.3 mA, respectively. In the field, groundwater contamination levels and required contaminant degradation rates are typically substantially lower than those applied in this study. Hence, actual electric energy consumption required to drive the bioelectrochemical oxidation process may be lower. Clearly, this scenario may change in case of groundwater characterized by conductivity values substantially lower than those used in this study (i.e. ≈3.2 mS cm^−1^), which, however, falls within the range of values typically reported for contaminated groundwater (i.e. 0.67–7.98 mS cm^−1^) (Naudet *et al*., 2004).

Notably, the fact that cell voltage did not substantially increase is also a straightforward indication that the electrocatalytic activity of the stainless steel cathode did not deteriorate over time, as it happens in some cases due to the precipitation of salts (e.g. calcium carbonate) or to corrosion phenomena.

**Figure 3:**
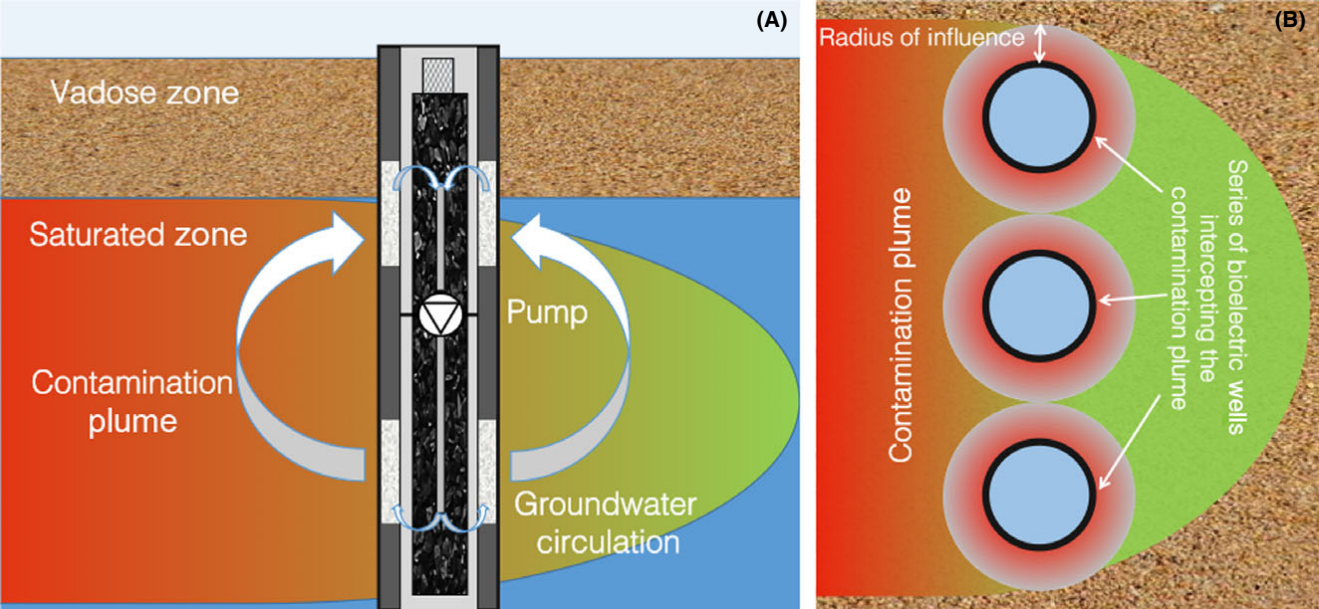
Cross‐sectional (A) and plan (B) view of an *in situ* groundwater bioremediation system based on the bioelectric well concept.

Finally, it should also be considered that the bioelectric well concept could be easily integrated within existing groundwater well designs and bioremediation schemes (Fig. 3A).

As an example, groundwater wells such as those based on the Groundwater Circulation Well^®^ technology (IEG, Germany), which are specifically designed to create a circulation flow of groundwater within the aquifer with the scope to mobilize contaminants and/or accelerate (anaerobic) biodegradation processes, are already commercially available (Pierro *et al*., 2017). In these systems, the contaminated groundwater is recirculated several times within the well before it flows downstream, hence allowing to control the groundwater retention time within the treatment zone independently of the groundwater flow velocity. The ‘capture zone’ of the well and, in turn, its radius of influence can be accordingly manipulated by changing the groundwater recirculation flow conditions and the size and positions of well screens. Along this line, a row of suitably spaced bioelectric wells can be employed to intercept and treat a contamination plume (Fig. 3B) or possibly to mobilize and treat a contamination source, thereby reducing its longevity. Necessarily, further studies are needed to optimize the technology with respect to a number of operational parameters (e.g. anode and cathode size and working potential, contaminant type and load rate, recycle flow rate). These efforts will eventually spur future field testing of this novel technology.

## Experimental

### The bioelectric well set‐up and operation

**Figure 4:**
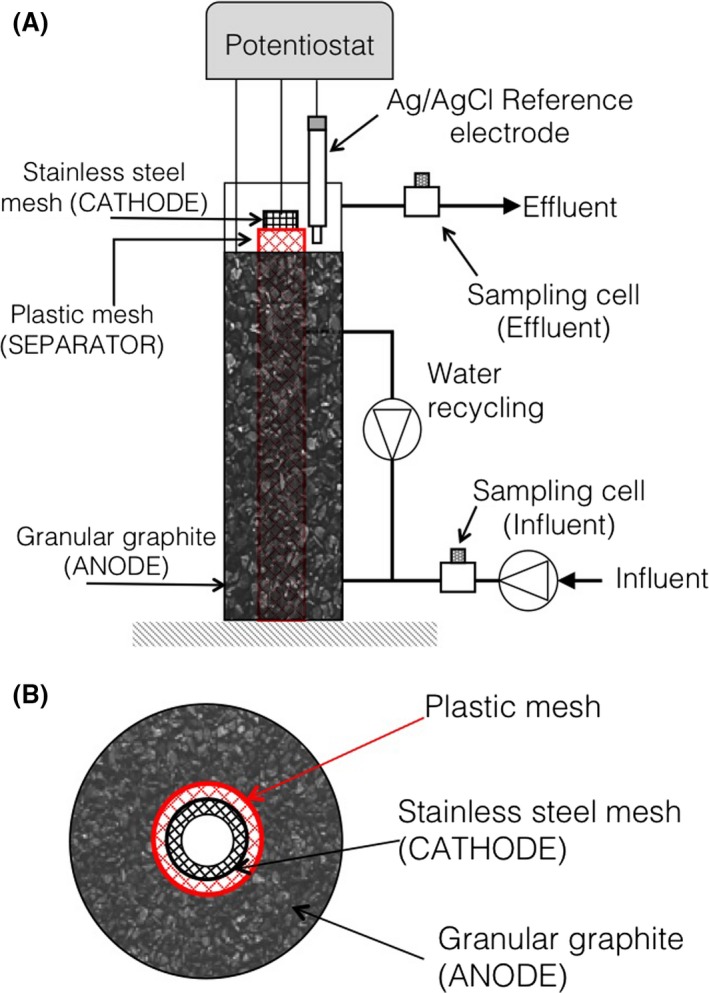
A. Schematic drawing of the laboratory‐scale scale experimental set‐up. B. Cross‐sectional view of the bioelectric well.

The laboratory‐scale prototype of the bioelectric well consisted of a 250 ml glass cylinder filled with graphite granules, serving as the bioanode, and housing a concentric stainless steel mesh (90 cm^2^ geometric surface area), serving as the cathode (Fig. 4).

Prior to use, the graphite granules were pretreated as described elsewhere (Gregory *et al*., 2004). A graphite rod (30 cm × 1.2 cm; Sigma‐Aldrich, Milan, Italy) was inserted into the bed of graphite granules and was connected to the external circuit by a titanium wire. A titanium wire was also used to connect the stainless steel cathode to the external circuit. The graphite anode was kept physically separated (yet hydraulically connected) by the stainless steel cathode by means of a polyethylene mesh (Fig. 4). An Ag/AgCl reference electrode (+198 mV versus SHE) was placed on top of the cylinder to control, by means of an IVIUMnSTAT potentiostat (IVIUM Technologies, The Netherlands), the potential of the bioanode at the desired value (i.e. +200 mV versus SHE). The glass cylinder was equipped with a number of ports positioned along its length. During operation, the system was continuously fed with synthetic contaminated groundwater, at a flow rate of 0.6 l d^−1^, through a port positioned at the bottom of the cylinder, while the treated groundwater was continuously discharged from a port positioned near the upper end of the cylinder. The synthetic groundwater used in the study contained (g l^−1^): NH_4_Cl (0.5), MgCl_2_·6H_2_O (0.10), K_2_HPO_4_ (0.4), CaCl_2_·6H_2_O (0.05) and 10 ml l^−1^ of a trace metal solution (Zeikus, 1977), and 1 ml l^−1^ of vitamin solution (Balch *et al*., 1979). Once prepared, it was flushed with a N_2_/CO_2_ (70:30 v/v) gas mixture in order to remove oxygen, the pH was adjusted at 7 by adding a NaHCO_3_ solution (10% w/v) and then it was transferred into a 2 l collapsible Tedlar gas bag, prior to being spiked with phenol to a final concentration of 25 mg l^−1^. During operation, the liquid phase within the glass column was continuously recycled at a flow rate of 75 ml min^−1^ to minimize the establishment of substrate, products and biomass concentration gradients. The average hydraulic retention time, estimated from a stepwise tracer (i.e. bromide) experiment, was 10.5 h. All tubings were made of Viton^®^ (Sigma‐Aldrich, Italy) to minimize adsorption of phenol and volatilization losses. The system was also equipped with two flow‐through, vigorously stirred sampling cells, each having a total volume of approximately 25 ml, which were placed at the inlet and at the outlet of the bioelectric well and which enabled sampling the influent and effluent liquid streams for analysis of phenol and pH, as well as the headspace for analysis of hydrogen, methane and oxygen. Throughout the study, the system was maintained at room temperature (i.e. 20 ± 3°C). At the start of the study, the bioelectric well was inoculated with 25 ml of a municipal activated sludge (Rome, Italy); on day 34, the system was bioaugmented with 25 ml of refinery wastewater from a petrochemical plant, as a potential source of phenol‐degrading microorganisms. System performance was primarily assessed in terms of phenol removal rate, electric current generation and coulombic efficiency.

### Amplification of 16S rRNA genes, sequencing and sequence analyses

At the end of the study, ≈20 g of graphite granules and 100 ml of liquid effluent were sampled from the bioelectric well and processed for microbial community analysis via next‐generation sequencing. The same analysis was carried out on a sample of the municipal activated sludge and of the refinery wastewater used to inoculate the reactor.

The genomic DNA was extracted using the FastDNA Spin Kit for Soil (MP Biomedicals, Solon, OH, USA) according to the manufacturer's instructions. Before DNA extraction, liquid samples were filtered on 0.22 μm polycarbonate filters and genomic DNA was extracted from the filters. The V5‐V6 hypervariable regions of the 16S rRNA gene were PCR‐amplified using the 783F and 1046R primers (Huber *et al*., 2007; Wang and Qian, 2009). PCR was performed as previously reported (Ferrentino *et al*., 2016). Amplicons were purified with the Wizard^®^ SV Gel and PCR Clean‐up System (Promega Corporation, Madison, WI, USA) according to the manufacturer's instructions and quantified using Qubit^®^ (Life Technologies, Carlsbad, CA, USA). Sequencing was performed at Parco Tecnologico Padano (Lodi, Italy) by MiSeq Illumina (Illumina, San Diego, CA, USA). Reads from sequencing were demultiplexed according to the internal barcodes. The UPARSE pipeline (Edgar, 2013) was used for the bioinformatics elaborations as previously reported (Daghio *et al*., 2016). Classification of the sequences representative of each OTU was performed using the RDP classifier (≥ 80% confidence) (Wang *et al*., 2007). The sequences were deposited in the European Nucleotide Archive with accession number PRJEB20095. The Shannon–Weiner index (*H*′) was calculated at the genus level to measure the α diversity. The unclassified sequences were not considered in the calculation of the microbial diversity.

### Analytical methods

Hydrogen, methane and oxygen were analysed by injecting 50 μl of headspace sample (with a gastight syringe) into a PerkinElmer GC Autosystem (2 m × 2 mm stainless steel column packed with 60/80 Carboxen 1000 Supelco; N_2_ carrier gas 20 ml min^−1^, oven temperature 150°C, injector temperature 200°C, thermal conductivity detector temperature 200°C).

Phenol was quantified by injecting 1 μl of liquid‐phase samples into a PerkinElmer GC 8500 gas chromatograph (2 m × 2 mm glass column, packed with 60/80 mesh Carbopak B/1% SP‐1000 Supelco; N_2_ carrier gas 18 ml min^−1^; oven temperature 210°C; flame ionization detector temperature 250°C).

### Chemicals

Phenol (99.5+%) was purchased from Sigma‐Aldrich (Italy). All of the other chemicals used to prepare analytical standard or feed solutions were of analytical grade and were used as received.

### Calculations

Phenol removal rate (*q*, mg l^−1^ d) was calculated according to the following equation:

(1) $$q=\frac{\left(C_{\mathrm{IN}}-C_{\mathrm{OUT}}\right)}{V_{\mathrm{BW}}}\times Q,$$

where *C*_IN_ and *C*_OUT_ (mg l^−1^) are the measured phenol influent and effluent concentrations; *Q* (l d^−1^) is the influent flow rate; and *V*_BW_ (l) is the total empty volume of the bioelectric well.

The coulombic efficiency (*η*, %) was calculated as the ratio between the measured electric current and the theoretical electric current which would be generated from the complete oxidation (to carbon dioxide and water) of the removed phenol, according to the following equation:

(2) $$\eta =\frac{i}{\frac{q\times V_{\mathrm{BW}}}{{\text{MW}}_{\mathrm{PHENOL}}}\times \frac{f}{24\times 3600}\times F}\times 100,$$

where *i* (mA) is the electric current flowing in the circuit; MW_PHENOL_ (mg mmol^−1^) is the molecular weight of phenol; *f* is the number of mmol of electrons released from the complete oxidation of 1 mmol of phenol; and *F* is the Faraday's constant.

## Conflict of interest

None declared.
